# Assessing Second Language Listening Over the Past Twenty Years: A Review Within the Socio-Cognitive Framework

**DOI:** 10.3389/fpsyg.2020.02123

**Published:** 2020-09-03

**Authors:** Lianzhen He, Ziyun Jiang

**Affiliations:** Institute of Applied Linguistics, Zhejiang University, Hangzhou, China

**Keywords:** second language listening assessment, socio-cognitive framework, listening comprehension, research theme, validity

## Abstract

The assessment of second language (L2) listening has received much attention. To understand the state-of-the-art research on L2 listening assessment, a total of 87 studies published in 14 peer-reviewed journals and two research report series between 2001 and 2020 were reviewed, using the socio-cognitive framework for developing and validating listening tests proposed by Weir (2005). Thirteen research themes were identified in relation to the six components of the framework, including test-taker characteristics, cognitive validity, context validity, scoring validity, consequential validity, and criterion-related validity. Context validity was the most investigated component, covering three research themes, that is, task setting, linguistic demands (input and output), and speakers. Based on a detailed analysis of the 13 research themes, recommendations for future research in L2 listening assessment were given.

## Introduction

Listening is the most frequently used mode of human communication, and “more than forty-five percent of our total communication time is spent in listening” (Feyten, 1991, p. 174). As one of the crucial components of successful human communication (Field, 2008; Rost, 2011), listening lies at “the heart of language learning” (Vandergrift, 2007, p. 191) and facilitates second language (L2) learning (Buck, 2018; Ockey and Wagner, 2018). As a multidimensional construct, listening consists of affective, behavioral, and cognitive processes (Halone et al., 1998; Worthington and Bodie, 2017). Assessing such a complex construct is challenging (Brindley, 1998; Buck, 2001, 2017; Vandergrift, 2007; Wagner, 2013b) and has become a focus of listening scholarship due to its significant role in education, politics, and society (Weir, 2013), with considerable efforts made to provide measures that are valid indicators of listening (Bodie and Worthington, 2017). Compared with listening in a first language (L1), L2 listening has more comprehension barriers which require L2 listeners to perform additional processes (Flowerdew and Miller, 2005).

Over the past 20 years, the field of L2 listening assessment has witnessed important development, and the importance of authenticity has been particularly underscored (Elliott and Wilson, 2013; Ockey and Wagner, 2018). An authentic assessment requires that the way test takers interact with the task corresponds to their use of language in the real-life communication contexts (Bachman and Palmer, 1996; Buck, 2001). As pointed out by Weir (2005, p. 98), “to test listening we must understand the processing that takes place in real-life situations and attempt to see that communication in our tests is anchored in the real world as far as possible.” The growing interest in authenticity has spurred research on the innovation of L2 listening assessment practices. For instance, large-scale standardized tests like the Test of English as a Foreign Language Internet-based Test (TOEFL iBT) were driven to embrace a wider view of listening (Weir and Vidakovic, 2013) and incorporate integrated tasks that involve listening and other skills (i.e., reading, speaking, and writing). Meanwhile, advances in computer technology have not only improved the quality of acoustic input in L2 listening assessment (Geranpayeh and Taylor, 2013) but also caused a surge of interest in the development and application of video-based listening (e.g., Wagner, 2010b), cognitive diagnostic assessment (e.g., Lee and Sawaki, 2009), computerized dynamic assessment (e.g., Poehner et al., 2015), and computerized adaptive testing (e.g., He and Min, 2017). These advances are evidenced by the increasing number of research articles published in peer-reviewed journals and research report series.

A handful of reviews on L2 listening assessment research have been conducted over the past two decades. Some discussed recent development and challenges in the field (e.g., Wagner, 2013b), and others focused on a specific theme of L2 listening assessment (e.g., Taylor and Geranpayeh, 2011). Taylor and Geranpayeh (2011) reviewed approaches to assessing listening for academic purposes. Drawing on the socio-cognitive framework (Weir, 2005), they focused on how to define and operationalize the construct of academic listening proficiency. These reviews provide helpful insights into the complex factors and challenges involved in L2 listening assessment. However, a comprehensive understanding of the state-of-the-art research in the field is still lacking, and it is unclear what research themes are important.

This study aims to give a comprehensive review of research on L2 listening assessment in journal articles and research reports published between 2001 and 2020 to facilitate the understanding of the state-of-the-art research in the field and to try to point out avenues for future research. As an influential theory of developing and validating language tests, the socio-cognitive framework (Weir, 2005; Geranpayeh and Taylor, 2013) was used to categorize research themes to make the review more coherent.

## The Socio-Cognitive Framework

The socio-cognitive framework (Weir, 2005) views the ability to be tested as the mental processes of test takers and conceives the use of language as a social rather than a purely linguistic phenomenon (Taylor, 2013). In relation to four macro skills of reading, listening, speaking, and writing, the framework has been widely used in a variety of contexts, especially in test development and validation projects. A typical example is its application in the validation of University of Cambridge ESOL Examinations (Shaw and Weir, 2007; Khalifa and Weir, 2009; Taylor, 2012; Geranpayeh and Taylor, 2013). Although the framework has been criticized for separating out many types of validity, which is a departure from Messick’s (1989) unitary theory of validity (Knoch and Chapelle, 2018), it presents a unified approach to conceptualizing and assembling different types of validity evidence in a comprehensive and coherent way (Taylor, 2013). In addition, it provides a transparent and plausible system for researchers and helps to analyze the key features of L2 listening assessment (Taylor and Geranpayeh, 2011, 2013). Therefore, it is considered suitable for the review of research on L2 listening assessment.

The framework contains six key components, namely test-taker characteristics, cognitive validity, context validity, scoring validity, consequential validity, and criterion-related validity (Weir, 2005). The first component is test-taker characteristics, which is divided into three types – physical/physiological characteristics, psychological characteristics, and experiential characteristics. Test-taker characteristics should be considered “at every stage of test development and continuously throughout live administrations of a test” (Taylor and Geranpayeh, 2013, p. 323). It is necessary that test developers attempt to design tests to elicit test-takers’ best performance through understanding test-taker characteristics and promoting feelings of comfort in test takers (Bachman and Palmer, 1996).

Related to test-takers’ cognitive or mental processing activated by the test task, the second component is cognitive validity, which addresses the extent to which test tasks require test takers to engage in cognitive processes that resemble those employed in a real-life listening situation (Field, 2013). Given that L2 listening involves a complex mechanism, the importance of understanding cognitive processes in L2 listening assessment has been underscored (Weir, 2005; Field, 2013). Drawing upon Cutler and Clifton (1999) model of L1 listening, Field (2013) presented a five-level processing model of L2 listening including input decoding, lexical search, parsing, meaning construction, and discourse construction, which can be divided into lower-level processing (i.e., input decoding, lexical search, and parsing) and higher-level processing (i.e., meaning construction and discourse construction).

The third component, context validity, concerns the contextual parameters of the test task, including linguistic content parameters and sociocultural contexts (Taylor, 2013), and is related to the extent to which test tasks are “representative of the larger universe of which the test is assumed to be a sample” (Weir, 2005, p. 19). Context validity is affected by multiple aspects, including task setting, administration, linguistic demands (task input and output), and speakers. These aspects are important to the development of tasks that are representative of the target language use (TLU) domain and the target language proficiency levels (Elliott and Wilson, 2013).

As the fourth component, scoring validity is related to the reliability of test scores and all aspects of the scoring process (Weir, 2005; Geranpayeh, 2013). The parameters of scoring validity include test difficulty, item bias, internal consistency, error of measurement, and grading and awarding. Developing valid items in terms of cognitive and contextual parameters matters little if student responses are not reported consistently (Taylor and Geranpayeh, 2013), so examination boards must devote considerable efforts to all aspects of scoring validity (Geranpayeh, 2013).

The fifth component, consequential validity, is concerned with test washback and impact and is closely related to fairness and ethics (Taylor, 2005; Hawkey, 2013). Test washback refers to the effect of tests on teaching and learning, and test impact is related to wider influences of tests in terms of educational systems and society in general (Hawkey, 2006, 2013). When tests are misused or abused, they can be viewed as unethical and unfair (Shohamy, 1997) and entail detrimental consequences for stakeholders (Bachman and Palmer, 2010). Therefore, it is important for test developers to consider the intended and unintended influences of tests (Bachman and Palmer, 2010).

The last component is criterion-related validity, including three aspects – comparison with different forms of the same test, cross-test comparability, and comparability with external standards and frameworks. Criterion-related validity is important because there would be no basis for meaningful score interpretation if different forms of a test are not comparable or tests which measure the same ability yield results that are not comparable to each other (Lim and Khalifa, 2013). In addition, it is necessary that the relationship between tests and external realities is consistently appropriate (Lim and Khalifa, 2013) because external standards and frameworks situate tests within larger contexts, which enhances the transparency and meaning of test results (Lim and Khalifa, 2013; Papageorgiou et al., 2019).

## Materials and Methods

Given the time and space limit, 14 peer-reviewed journals were targeted due to their relevance to the present study and the quality of the articles published in those journals. In addition, Educational Testing Service (ETS) and the International English Language Testing System (IELTS) research report series were included to provide a comprehensive picture of L2 listening assessment research. The two research report series were chosen because they include rigorous studies conducted by leading researchers from all over the world.

The articles and research reports were retrieved online *via* keyword search. Variations of the following terms were used in the search: *listening assessment*, *listening test*, and *listening task*. Two selection criteria were used in our examination of the titles and/or abstracts of the studies: (1) the study involved L2 test takers and focused on L2 listening assessment, or it investigated the assessment of multiple skills with specific discussion on L2 listening assessment and (2) the study was an empirical study or a systematic review. A total of 89 studies – 79 journal articles and 10 research reports – were initially retrieved. After careful reading of all the studies, two research reports were excluded because they had the same research design and used the same data with two journal articles included in the current study, resulting in a final dataset of 87 studies. Table 1 presents the number of studies included in the dataset for the current study.

**Table 1:** Number of articles taken from the 14 journals and two research report series.

Journal/research report series	Number of selected articles	%
Language Testing	20	22.99
Language Assessment Quarterly	14	16.09
System	11	12.64
TESOL Quarterly	7	8.05
Applied Linguistics	5	5.75
IELTS Research Report Series	4	4.60
Language Learning	4	4.60
ETS Research Report Series	4	4.60
Journal of English for Academic Purposes	3	3.45
Modern Language Journal	3	3.45
Studies in Second Language Acquisition	3	3.45
Computer Assisted Language Learning	2	2.30
Foreign Language Annals	2	2.30
Frontiers in Psychology	2	2.30
Language Learning and Technology	2	2.30
Journal of Educational Research	1	1.15
Total	87	100

Table 2 presents a coding scheme based on the socio-cognitive framework (Weir, 2005; Geranpayeh and Taylor, 2013). The coding was done manually. First, the two authors read each study carefully and coded it independently. Some studies were coded into more than one category since they investigated multiple components of the socio-cognitive framework. The initial intercoder agreement was high, reaching 89.66%. Incongruence between the coding results was discussed between the authors, and another expert in the field was invited if the incongruence remained unresolved. For instance, the authors disagreed on the coding of Wei and Low (2017), a study on test-takers’ score change pattern and increase rate. After discussion with the expert, the authors agreed that this study should be coded into comparison with different forms of the same test under criterion-related validity.

**Table 2:** The coding scheme based on the socio-cognitive framework.

Components		Research themes
Test-taker characteristics	1	Physical/physiological characteristics
	2	Psychological characteristics
	3	Experiential characteristics
Cognitive validity	4	Cognitive processes
Context validity	5	Task setting
	6	Setting: administration
	7	Linguistic demands (task input and output)
	8	Speakers
Scoring validity	9	Test difficulty
	10	Item bias
	11	Internal consistency
	12	Error of measurement
	13	Grading and awarding
Consequential validity	14	Washback on individuals in classroom/workplace
	15	Impact on institution and society
Criterion-related validity	16	Comparison with different forms of the same test
	17	Cross test comparability
	18	Comparability with external standards and frameworks

## Results

Five out of the 18 research themes in the coding scheme were not addressed in our dataset, that is, administration, test difficulty, error of measurement, impact on institution and society, and comparison with different forms of the same test. Therefore, only 13 research themes were identified, as is shown in Table 3. Among the six components, context validity was the most investigated (*N* = 57, 65.52%), followed by test-taker characteristics (*N* = 21, 24.14%), cognitive validity (*N* = 12, 13.79%), scoring validity (*N* = 8, 9.2%), criterion-related validity (*N* = 4, 4.6%), and consequential validity (*N* = 1, 1.15%). And among the 13 research themes identified, task setting (*N* = 34, 39.08%) was the most investigated, followed by linguistic demands (task input and output; *N* = 14, 16.09%) and cognitive processes (*N* = 12, 13.79%). The 13 research themes will be discussed in detail in the following sections.

**Table 3:** Summary of research themes based on the socio-cognitive framework.

Components		Research themes	Number of articles (%)
Test-taker characteristics	1	Physical/physiological characteristics	7 (8.05)
	2	Psychological characteristics	13 (14.94)
	3	Experiential characteristics	1 (1.15)
Cognitive validity	4	Cognitive processes	12 (13.79)
Context validity	5	Task setting	34 (39.08)
	6	Linguistic demands (task input and output)	14 (16.09)
	7	Speakers	9 (10.34)
Scoring validity	8	Item bias	5 (5.75)
	9	Internal consistency	2 (2.3)
	10	Grading and awarding	1 (1.15)
Consequential validity	11	Washback on individuals in classroom/workplace	1 (1.15)
Criterion-related validity	12	Comparison with different forms of the same test	1 (1.15)
	13	Comparability with external standards and frameworks	3 (3.45)

Fifteen studies (17.24%) were coded into multiple research themes, with 14 (16.09%) coded into two themes and one (1.15%) into three themes.

### Test-Taker Characteristics

#### Physical/Physiological Characteristics

Physical/physiological characteristics cover obvious biological features shared by test takers like gender and age, short-term ailments like a heavy cold, and long-term disabilities such as dyslexia (O’Sullivan, 2000; Weir, 2005; Elliott, 2013). A common approach to investigating physical/physiological characteristics is differential item functioning (DIF) analysis, which is used to detect the variation of responses across different subgroups of test takers. DIF exists when the probability of answering one item correctly differs for subgroups of test takers with comparable ability (Min and He, 2020). Geranpayeh and Kunnan (2007) conducted bias analyses of listening test items of the Certificate in Advanced English examination in terms of age. In their study, test takers were divided into three age groups (i.e., 17 and younger, 18–22, and 23 and older). Although they reported that no age group was clearly disadvantaged, it was observed that the 17 and younger group performed worse than the other two groups. One possible reason was that the test topics were less attractive to younger test takers.

Similarly, researchers investigated whether DIF existed across gender subgroups in listening tests, and gender-based DIF was detected (Park, 2008; Aryadoust et al., 2011). Conducting DIF analysis of the Michigan English Language Assessment Battery (MELAB) listening test, Aryadoust et al. (2011) observed that males with lower listening proficiency were likely to score higher on some items than females and males with higher listening proficiency. Apart from exploring test-takers’ responses, recent studies probed into the gender effect in test preparation and test-taking processes. For instance, Chou (2019) investigated whether gender predicted self-efficacy in test preparation for the listening section of the University Entrance Examination test in Taiwan and reported that gender was not associated with self-efficacy, test anxiety, and strategy use. Moreover, Aryadoust et al. (2020) conducted a neuroimaging study and employed functional near-infrared spectroscopy (fNIRS) to uncover the test-takers’ neurocognitive mechanisms involved in listening tests. They observed differences in neural substrates across genders, although differences in the test scores of males and females were not statistically significant.

In addition to age and gender, research interest in dyslexia has emerged. Dyslexia is one of the most common learning difficulties test takers have and is categorized into physical/physiological characteristics together with other long-term illnesses or disabilities such as speech defects (O’Sullivan, 2000; Weir, 2005; Elliott, 2013). Dyslexic learners are characterized by the “underlying weakness in the areas of working memory, executive functioning, and processing speed” (Kormos et al., 2019, p. 835). In Kormos et al.’s (2019) study, the listening test performance of young dyslexic and non-dyslexic learners was compared, and dyslexic test takers performed worse than their non-dyslexic peers. In some countries, there is a legal requirement that test takers with specific learning difficulties such as dyslexia should be accommodated (Weir, 2005). However, it is controversial as to what special arrangements should be offered to test takers to make tests assess abilities rather than disabilities, ensuring fair tests for every test taker without compromising test validity is challenging to test developers (Kosak-Babuder et al., 2019).

#### Psychological Characteristics

Psychological characteristics include cognitive characteristics such as memory and affective characteristics like motivation (Elliott, 2013). Four psychological characteristics have received much research attention, including working memory, metacognition, motivation, and anxiety. Working memory is the ability to “keep track of ongoing mental processes and moment-to-moment changes in the immediate environment” (Logie, 2011, p. 240) and is essential for complex cognitive activities (Olive, 2004). Brunfaut and Revesz (2015) investigated the correlation between test-takers’ performance on working memory tasks and 11 listening tasks of Pearson Test of English Academic (PTE Academic). Results showed that test-takers’ listening scores were positively correlated with their working memory capacity, and listening tasks assessing local comprehension (i.e., listening for specific details) put higher demands on working memory than those assessing global comprehension (i.e., listening for main ideas).

Metacognition refers to learners’ ability to control their thoughts and regulate their own learning (Vandergrift and Goh, 2012), which plays an important role in learning to listen (Vandergrift and Goh, 2012). Researchers have investigated test-takers’ use of metacognitive strategies, such as planning for, monitoring, and evaluating listening. More specifically, Wang and Treffers-Daller (2017) used Metacognitive Awareness Listening Questionnaire (Vandergrift et al., 2006) to measure the effect of metacognition on the listening scores of College English Test Band 4 (CET 4). A significant positive correlation between test-takers’ listening scores and metacognitive awareness was reported, although it was relatively low (*r* = 0.19), compared with test-takers’ vocabulary size (*r* = 0.44) and general language proficiency (*r* = 0.36).

Closely related to metacognition, motivation is a continuum consisting of amotivation, extrinsic motivation, and intrinsic motivation in self-determination theory (Deci and Ryan, 1985, 1995). Drawing on this theory, Vandergrift (2005) provided empirical evidence for the interplay between motivation and metacognition and for their effect on listening scores. In his study, a greater use of metacognitive strategies was related to a higher level of motivation. Moreover, test-takers’ listening scores were correlated negatively with amotivation, while a high level of motivation did not appear to be a reliable predictor of L2 listening proficiency. Another study on motivation was conducted by Xu (2017), who used expectancy-value theory (Wigfield and Eccles, 2000) to conceptualize test-taking motivation. He observed the mediating effect of metacognition on the relationship between motivation and the listening scores of CET 4. The findings revealed that the effect of motivation on listening scores was pronounced, and increased listening metacognitive awareness improved test-takers’ listening performance when their motivation level was stable.

Anxiety is another important psychological characteristic explored in our dataset. Foreign language listening anxiety has received some attention, which is the type of anxiety experienced by learners in the listening context, and consists of communication apprehension, test anxiety, and fear of negative evaluation (Horwitz et al., 1986). The negative effect of foreign language listening anxiety was observed by Zhang (2013), who investigated the causal relations between foreign language listening anxiety and IELTS listening test scores and found that anxiety negatively affected test-takers’ performance on the IELTS listening test. This negative effect was also observed by Brunfaut and Revesz (2015) who reported that less anxious test takers performed better on the listening section of PTE Academic. Instead of focusing on foreign language listening anxiety, In’nami (2006) explored the relationship between test-takers’ test anxiety and performance in familiar listening tasks (i.e., multiple choice questions and open-ended questions) and found that test anxiety did not influence test performance, suggesting that test anxiety can be independent of the other two components of foreign language listening anxiety (i.e., communication apprehension and fear of negative evaluation).

#### Experiential Characteristics

Experiential characteristics concern test-takers’ experience in preparing and taking tests and their familiarity with the test, including test-takers’ educational and cultural background (Elliott, 2013). The effect of test-takers’ preparation on their IELTS listening test scores was investigated by Winke and Lim (2017), who explored the effects of listening test preparation on listening scores, test-taking strategies, and anxiety. Three types of instruction were given in their study, that is, explicit preparation (i.e., test-taking-strategies instruction and practice tests), implicit preparation (i.e., vocabulary instruction and practice tests), and conversation classes plus a practice test. They found that all of the three types of instruction helped test takers perform better in listening tests, while there were no differential effects on scores, strategy use, or anxiety levels among the three types. They concluded that concise test preparation (i.e., one simple practice test) helped test takers perform better, and extensive test preparation lasting months or years might not be necessary.

### Cognitive Validity

It is common that listening is assessed as a composite of several subskills (Worthington, 2017). Listening subskills reflect core cognitive processes measured in L2 listening tests, and researchers have not reached consensus on what subskills make up L2 listening. A popular approach to investigating listening subskills is the use of cognitive diagnosis models. Listening subskills were found to be different in terms of various grain sizes (Sawaki et al., 2009), and the contribution of a particular listening subskill was not consistent across items (Yi, 2017), indicating the vague definition of L2 listening subskills (Aryadoust, 2020). To address this gap, Aryadoust (2020) used the document co-citation analysis to give a systematic review of research on comprehension subskills. An integrative framework of comprehension subskills was provided, which included a total of 18 L2 comprehension subskills.

In addition to listening subskills, items targeting different levels of listening comprehension, such as local (i.e., explicit and factual) and global (i.e., inferential) comprehension, have been investigated. For instance, Becker (2016) examined the extent to which the two types of items differentiated between test takers with different proficiency levels. Since items targeting different levels of listening comprehension were able to distinguish different proficiency groups, and items targeting local comprehension were easier than those targeting global comprehension for all groups, Becker provided empirical evidence for the hierarchy of cognitive processes and the relative difficulty of items targeting different cognitive processes.

A variety of methods were used to probe into test-takers’ cognitive processes, such as stimulated recall protocols, questionnaires, content analysis, and advanced technology. One typical example is Field (2009), who investigated the cognitive validity of a lecture-based note-taking task in the IELTS listening test by comparing the cognitive processes of participants under test and non-test conditions. Evidence in the verbal report revealed that cognitive processes under the two conditions were incongruent. More precisely, participants adopted test-wise strategies under test conditions. Also, the processing of many participants was superficial under test conditions as they focused on lexical matches instead of the overall meaning. Carrell (2007) focused on test-takers’ note-taking behavior on academic lecture tasks consisting of multiple-choice questions. A significant correlation between content words in the notes and listening scores was observed and test takers tended to write down content words following the linear order of the lectures instead of using abbreviations, symbols, or paraphrasing. Carrell’s study contributed to the understanding of the content and quality of test-takers’ notes in L2 listening assessment.

Instead of focusing on tasks that only require listening, Rukthong and Brunfaut (2019) explored the cognitive processes involved in an integrated task (i.e., a listening-to-summarize task). With an increasing popularity, integrated tasks require test takers to complete tasks employing at least two language skills (Rukthong and Brunfaut, 2019) and have been acclaimed for authenticity (Wagner, 2013b) as well as positive washback (Taylor and Geranpayeh, 2011). Based on data collected from a stimulated recall protocol and perception questionnaire, they found that test takers relied on various listening processes, including both higher-level and lower-level processing. The cognitive processes of listening play a crucial role in completing integrated tasks which involve listening.

Advanced technology has been employed in the investigation of cognitive processes, including eye-tracking technology (Suvorov, 2015; Holzknecht et al., 2020) and neuroimaging (Aryadoust et al., 2020). Test-takers’ eye movement during the listening test can be recorded by eye-tracking technology to understand their oculomotor engagement with test items, such as the stems and options of multiple-choice questions. For instance, Suvorov (2015) recorded test-takers’ eye movement during the video-based listening test including context and content videos, and no significant difference was observed in test-takers’ oculomotor engagement with content and context videos. More recently, Holzknecht et al. (2020) observed that test takers paid significantly less attention to later options when answering listening items from the Aptis Test using eye-tracking technology. Aryadoust et al. (2020) investigated brain activation patterns under test conditions using functional magnetic resonance imaging (fMRI). Among the main techniques of understanding how different parts of the brain are engaged in psychological and behavioral functions (Burunat and Brattico, 2017), fMRI has been used by neuroscientists and physicians and was first applied to L2 listening assessment by Aryadoust et al. They introduced the notion of neurocognitive validity, which means that a listening test should engage the neurocognitive processes which are required in real-life contexts. The use of advanced technology has provided deeper insights into cognitive processes, which may have implications for test development and validation.

### Context Validity

#### Task Setting

Task setting is the most investigated research theme in our dataset, which is not surprising due to the important role of task characteristics in L2 listening assessment. A wide range of task setting parameters have been investigated, and the complexity of interactions between these parameters was observed (Brindley and Slatyer, 2002; Brunfaut and Revesz, 2015). Four aspects of task setting received much attention, that is, task purpose and rubric, response method, modality/channel of presentation, and time constraints.

Five studies in our dataset have explored task purpose and rubric. Researchers have investigated listening tasks that are developed for assessing translanguage and those for assessing pragmatic competence. Specifically, Baker and Hope (2019) developed a translanguaged French/English listening task for university professors. In their study, text types were chosen from the TLU domain, including short telephone messages, an introduction and biography of a guest speaker, and a departmental meeting. Also, listening scripts were developed based on the recordings of authentic departmental meeting to incorporate authentic syntactic and discourse functions into the task. In addition to translanguaged listening tasks, pragmatic listening tasks were developed to assess test-takers’ ability to comprehend speakers’ intentions (Taguchi, 2005, 2007, 2008a,b). Taguchi (2005) incorporated dialogues with the interactive characteristics of spoken English, such as discourse markers, interjections, or hesitation markers, and Taguchi (2008b) gleaned linguistic features from the synthesis of a literature review, survey, and field notes, tapping into different types of implied meaning.

Second, researchers had much interest in response methods, with a particular focus on multiple-choice questions, open-ended questions, partial dictation, and note-taking tasks. A given response method only tests part of the listening construct, and over-reliance on a single response method may lead to construct under-representation (Elliott and Wilson, 2013). Therefore, it is generally desirable to use various response methods in listening assessment (Khalifa and Weir, 2009). For example, 11 different response methods are employed in the listening section of PTE Academic, which are designed to assess a wide range of listening skills (Wei and Zheng, 2017).

As a mainstay of listening assessment, multiple-choice questions provide retrieval cues which facilitate recall of information from the listening input (Chung, 2002). The prevalence of multiple-choice questions could be attributed to practical benefits such as grading and editing (Elliott and Wilson, 2013). Many issues related to multiple-choice questions have been investigated, including the effect of item preview (Chang and Read, 2006; Yanagawa and Green, 2008; Koyama et al., 2016), the mode of presenting items (Chang and Read, 2013), the language of questions (Filipi, 2012), the number of options (Lee and Winke, 2013), and response order (Holzknecht et al., 2020).

Different from multiple-choice questions, open-ended questions, partial dictation, and note-taking tasks are constructed response formats, which require test takers to formulate their own answers with words or phrases and can effectively evaluate test-takers’ listening and their ability to reconstruct what they have heard (Cheng H., 2004). Researchers compared open-ended questions with multiple-choice questions and found that test takers performed better on multiple-choice questions (Chung, 2002; Cheng H., 2004; In’nami and Koizumi, 2009). Targeting partial dictation tasks, Cai (2013) investigated the difficulty and internal consistency of phrasal and single-word partial dictation tasks and found that the two types of partial dictation tasks were comparable. In terms of note-taking tasks, the outline format and blank format of note-taking tasks were explored in Song (2012), who found that note quality indices, especially the number of topical ideas and the organization of notes, were good indicators of listening proficiency, and the outline format was a more reliable measure of L2 academic listening than the blank format.

Third, 14 studies explored modality/channel of presentation, with a particular focus on the use of visual input, such as images and videos. Although the use of visual input is an important aspect of promoting authenticity, whether to use visuals in listening assessment remains open for discussion (Kellerman, 1992; Gruba, 1997; Buck, 2001; Taylor and Geranpayeh, 2011; Wagner and Ockey, 2018). Allowing test takers to employ visual input in understanding the aural input tends to bring about construct-irrelevant variance. Traditionally, L2 listening assessment is “typically concerned with mastery of the language itself, not that of pancultural, *ad-hoc*, gesture-based communication” (Batty, 2015, p. 17). However, trying to separate the effect of visuals from audio elements is unproductive (Gruba, 1997). Most real-life listening involves visual input which aids in comprehension, and various channels are employed by listeners to construct the meaning of what they are hearing (Gruba, 2004, 2006) and videos have become an important part of the listening construct due to the technological advances.

Research on the role of videos in L2 listening tests produced mixed results. Non-verbal information in videos was found to improve test scores (Ginther, 2002; Jones and Plass, 2002; Sueyoshi and Hardison, 2005; Wagner, 2010b, 2013a; Dahl and Ludvigsen, 2014). However, the score difference was not pronounced (Coniam, 2001; Cubilo and Winke, 2013; Batty, 2015; Suvorov, 2015). Using the Rasch model, Batty (2015) found that the difference in item difficulty of video-based and audio-only tasks was small. Test takers varied in their attitudes toward videos, some interacting extensively with videos and preferring video-based tasks to audio-only tasks (Sueyoshi and Hardison, 2005; Ockey, 2007; Wagner, 2007, 2008, 2010a; Cubilo and Winke, 2013), while others reporting that visuals were distracting (Coniam, 2001).

Lastly, as an important aspect of context validity, time constraints have been explored. In L2 listening teaching and assessment practices, the input is sometimes repeated to make the information more comprehensible. However, second hearings are often not possible in the TLU domain, and once-heard texts have greater authenticity (Taylor and Geranpayeh, 2011). Elkhafaifi (2005) found that the repeated exposure to the listening passage improved test-takers’ performance, concurring with findings of other studies (Brindley and Slatyer, 2002; Sakai, 2009; Holzknecht et al., 2020). Sakai (2009) divided test takers into two listening proficiency groups according to their pretest scores and explored the interactional effect between repetition and proficiency levels. Their performance on the free written recall tasks in the first and second hearing conditions was compared. Results showed that the repetition of listening passages led to more precise comprehension and was effective for both proficiency groups.

#### Linguistic Demands (Task Input and Output)

In terms of linguistic demands, the type of input texts (i.e., monologic/dialogic texts and scripted/unscripted texts) has received much research interest. For instance, Read (2002) found that a monologue was significantly easier than a dialogue of the same content. Papageorgiou et al. (2012) examined the difference between monologic and dialogic texts through statistical and content analyses. They found that monologues, compared with dialogues, were more structured and contained additional explicit statements, and the relative difficulty of monologic and dialogic texts varied across items. Apart from monologues and dialogues, unplanned informal conversations and formal written language have been compared. The inclusion of unscripted texts is considered to be more authentic (Wagner, 2013b) and more challenging (Read, 2002; Wagner and Toth, 2014), probably because test takers are more familiar with scripted texts than unscripted texts (Read, 2002) and the spoken input learners hear often consists of textbook texts which lack the characteristics of the unplanned discourse mode (Wagner and Toth, 2014).

Another line of research focused on the role of lexical and grammatical resources in L2 listening tests. The relative importance of lexical and syntactic knowledge in L2 listening test was investigated. It was found that both lexical and syntactic resources played an important role in successful L2 listening, and the role of lexical resources was more important than that of syntactic resources (Cai, 2020; Vafaee and Suzuki, 2020). Furthermore, empirical evidence showed that vocabulary knowledge is a strong predictor of L2 listening performance (Andringa et al., 2012; Matthews and Cheng, 2015; Wang and Treffers-Daller, 2017). Staehr (2009) investigated the depth and breadth of test-takers’ vocabulary knowledge and their listening performance and found that a lexical coverage of 98% was needed in the listening test. In van Zeeland and Schmitt (2013) study, most L2 participants understood everyday narrative texts with a lexical coverage of 90–95%. More recently, researchers have explored the effect of aural vocabulary knowledge (Cheng and Matthews, 2018; Matthews, 2018; Li, 2019), which refers to the knowledge of words mediated through the aural modality (Matthews, 2018). A significant positive correlational relationship between test-takers’ aural vocabulary size and listening scores was found (Matthews, 2018; Li, 2019).

In addition, the lexical complexity of listening passages has garnered much research attention. Brunfaut and Revesz (2015) found that the lexical complexity of listening input was significantly correlated with item difficulty. They reported that listening passages including low-frequency phrases were significantly more difficult. However, Paribakht and Webb (2016) did not find any correlation between the lexical coverage of academic words in listening passages and test-takers’ listening performance. One possible reason was that other factors such as test-takers’ strategy use and content knowledge will impact the outcomes.

#### Speakers

With the diversity of accents that English speakers are exposed to in the TLU domain for which many listening tests are designed (Taylor and Geranpayeh, 2011), L2 listening assessment has been argued to reveal the changing demographics in English speaking contexts (Ockey and French, 2014) by incorporating accented speech. For example, inner and outer circle English accents have been used in high-stakes listening tests, including the TOEFL iBT, Test of English for International Communication (TOEIC), and IELTS (Kang et al., 2019). However, concerns about the inclusion of non-standard accents have been raised. According to the interlanguage speech intelligibility benefit (Bent and Bradlow, 2003), also called a shared-L1 advantage phenomenon, test takers who share the same L1 with the speakers of listening passages can understand listening materials more easily. If the inclusion of non-standard accents results in a subgroup of test takers being advantaged, using non-standard accents may introduce construct-irrelevant variance (Elliott and Wilson, 2013) and have detrimental effects on test fairness.

Empirical evidence provided partial support for a shared-L1 advantage phenomenon (Major et al., 2002; Harding, 2012; Dai and Roever, 2019; Kang, et al., 2019). Major et al. (2002) found that Spanish-L1 test takers scored higher when listening to Spanish-accented speech, but Chinese-L1 test takers performed worse when listening to Chinese-accented speech. However, Harding (2012) observed that Chinese-L1 test takers were advantaged on Chinese-accented items, while the facilitative effect of L1 accents was not clearly observed in the group of Japanese-L1 test takers. Dai and Roever (2019) divided Chinese-L1 adolescent test-takers into four groups, each of which took one accented version of the same English listening test. Results showed that the Chinese-accented group scored highest, followed by the Spanish, Australian, and Vietnamese-accented groups. Additionally, the beneficial shared-L1 effect was strongest for gap completion items, indicating the highly complex interplay between the effect of accents and task types. Kang et al. (2019) found that Indian-L1 and South African-L1 test takers benefited from their own accent, but they did not observe the shared-L1 effect on test scores because test takers performed significantly better when listening to standard American or British English.

In addition, the effect of accent strength and familiarity has been investigated (Matsuura et al., 2014; Ockey and French, 2014). Ockey and French (2014) developed a strength of accent scale based on salience and comprehensibility and a survey assessing test-takers’ familiarity with accents. They found that listening scores decreased as strength of accent increased and familiarity with accents was an advantage for test takers. Likewise, Matsuura et al. (2014) found that L2 listeners performed worse when listening to nonnative English speech, and less familiar accent was more difficult than a more familiar one.

Another line of research focused on the intelligibility of accents (Kang et al., 2018a,b, 2020). Intelligibility refers to the extent to which the speakers’ intended utterance is understood by listeners, which is generally measured by transcription tasks (Kang et al., 2018a,b). Kang et al. (2018b) examined the relationship between the phonetic/phonological features of speakers and intelligibility, which helps test developers to select speakers with different English accents for listening input. More recently, Kang et al. (2020) examined the relationship between test-takers’ proficiency levels and comprehension of different accents. They found that test-taker’s proficiency levels affected their comprehension of accented speech, and the performance of intermediate-level test takers, whose TOEIC scores were between 305 and 400 (i.e., 61–80th percentile), was more sensitive to speech with different accents than the beginner and advanced groups.

### Scoring Validity

#### Item Bias

One important aspect of scoring validity is that test results are free from bias (Weir, 2005). A test may be considered biased when there is systematically differential performance among subgroups of test takers with the same ability (Geranpayeh, 2013). Four studies in the dataset examined if test results biased toward a subgroup of test takers in terms of their L1 background (Harding, 2012), gender (Park, 2008; Aryadoust et al., 2011), and age (Geranpayeh and Kunnan, 2007). In addition, Batty (2015) conducted differential distractor functioning (DDF) analysis, similar to DIF analysis, to examine if test takers interacted with a particular distractor in video-based and audio-only multiple-choice questions. Batty found that one item revealed significant DDF, and it was difficult to explain the sources of DDF. Although research on item bias provides information about potential sources of bias and contributed to a better understanding of score-based decisions (Min and He, 2020), it is challenging to identify the reasons for items exhibiting significant DIF (Geranpayeh and Kunnan, 2007; Batty, 2015).

#### Internal Consistency

As a key parameter of scoring validity, internal consistency contains many aspects, including internal consistency coefficients, composite reliability, marker reliability, G-theory, and Item Response Theory (IRT)-based reliability (Geranpayeh, 2013; Geranpayeh and Taylor, 2013). IRT or Rasch models have been widely used to investigate internal consistency. For instance, IRT analyses were conducted to estimate the internal consistency for the listening scores across different groups of test takers and across different items (Pardo-Ballester, 2010).

Widely used in L2 listening assessment, testlets refer to sets of items that are based on the same input (Eckes, 2014). Testlets tap into higher-level skills and make item writing and test administration more efficient; however, items nested within testlets might violate one of the assumptions of IRT models, that is, the local independence assumption (Eckes, 2014). This assumption is maintained if a person’s response to an item does not affect the probability of the person’s response to another item (Eckes, 2014). As testlets may have negative influence on the precision of ability estimates and test reliability, Eckes (2014) examined the testlet effect of the listening section of the Test of German as a Foreign Language (TestDaF) and observed small or moderate testlet effects. Eckes compared different approaches of analyzing testlet-based tests, including the use of independent-items models, the polytomous-items model, and the testlet response theory (TRT; Wainer et al., 2007) model. Eckes found that treating testlet items as independent items (i.e., the use of independent-items models) or as a single polytomous superitem (i.e., using the polytomous IRT model) led to the inaccurate estimation of test reliability and test-takers’ ability.

#### Grading and Awarding

Listening tests often consist of multiple components targeting different communication goals (Choi and Papageorgiou, 2020). Scores on each component of the listening test, also called listening subscores, may provide added value over the total score. To examine the justifiability of reporting subscores at the individual and school levels, Choi and Papageorgiou (2020) explored the reliability and distinctiveness of listening and reading subscores of the TOEFL Primary test. Four listening subscores based on different communication goals were targeted, that is, Monologue, Dialogue, Narrative, and Academic subscores. They found that the individual-level subscores lacked psychometric added value, while the school-level subscores provided fine-grained information about the strengths and weaknesses of test takers from different schools, indicating that it is necessary to consider in score reporting what is reported and who is the intended user.

### Consequential Validity

One study in our dataset explored consequential validity, focusing on washback (Nguyen and Gu, 2020). The researchers investigated the washback of the TOEIC listening and reading tests, which were used as an exit requirement, on teaching in Vietnam. Moreover, to understand the mechanism of washback, they explored three types of factors in washback – test factors, personal factors, and context factors. They found that teachers tended to tailor their teaching content and methods to the demands of the test by focusing on the tested skills while devoting less time to communicative activities. In relation to the mechanism of washback, test and personal factors played a significant role and influenced teachers’ tendency to teach to the test and their use of communicative activities. In comparison, context factors were not closely related to the perceived washback. They argued that washback of the TOEIC in the Vietnamese context had not been fully understood and follow-up studies were needed to elucidate the reasons why these factors were correlated with washback.

### Criterion-Related Validity

#### Comparison With Different Forms of the Same Test

As the only study on the comparability of test forms, Wei and Low (2017) examined the longitudinal score change pattern of 19,855 repeaters – test takers who took the test six times in 68 administrations over a period of 4 years – by analyzing the scores of the monthly administered TOEIC listening and reading tests. The starting month and the spacing of the six test-taking months varied across the repeaters. Linear growth modeling results showed that the repeaters’ scores were stable over time (i.e., months) as their monthly score increases were small (i.e., a 1.6 score point increase per month), suggesting a high reliability of test scores across forms and across administrations. They also found that test scores varied much more between test takers than they varied overtime within test takers, and test-takers’ background variables, especially gender, educational levels, and test-taking experience, had impacts on their listening score growth patterns and increase rate.

#### Comparability With External Standards and Frameworks

Three studies have explored the comparability between listening tests and criteria measures, including academic lecture tasks (Sawaki and Nissan, 2009), final grades in degree courses (Breeze and Miller, 2011), and local tests (Wagner, 2016). Since TOEFL iBT can be interpreted as a measure of academic listening ability (Sawaki and Nissan, 2009), it is important to gather empirical evidence about the relationship between TOEFL iBT listening test and an appropriate criterion measure of academic listening. Sawaki and Nissan (2009) investigated the relationship between test-takers’ performance on TOEFL iBT listening test and academic lecture tasks that L2 English speakers encounter in their daily academic life. The researchers found that the listening test scores and the results of the criterion measure were positively correlated, indicating that they measured a similar academic listening construct.

Scores on large-scale L2 proficiency tests like TOEFL iBT and IELTS are used for many purposes, such as admission, placement, and exit. Breeze and Miller (2011) investigated the predictive validity of IELTS listening test as an entry requirement for admission to degree courses taught partly in English in a Spanish university. They found that test-takers’ listening test scores were correlated with their final grades in programs in Humanities, Law, and Medicine, which justified the use of IELTS listening test for admission to academic programs. To be noted, IELTS listening test scores only accounted for a small part of academic success, which was not surprising given that aspects other than listening ability may determine students’ academic success.

Research on the comparability with external standards and frameworks not only justifies the use of L2 listening tests but also helps score users to make better decisions. Specifically, Wagner (2016) investigated the use of TOEFL iBT speaking and listening tests for international teaching assistants (ITAs) screening purposes. Three criteria measures of ITAs’ language proficiency and teaching competence were included in his study, that is, the SPEAK test assessing ITA’s oral proficiency, the TEACH test that measured ITAs’ mastery of the curriculum, and undergraduate students’ evaluations of their ITAs’ language proficiency and teaching competence. TOEFL iBT listening test scores had significant correlations with the criteria measures. More importantly, TOEFL iBT listening test scores predicted ITAs’ teaching competence better than TOEFL iBT speaking test scores, as the listening test scores accounted for an additional 15.3% of the variance of students’ assessment of ITAs’ teaching competence, whereas the speaking test scores accounted for only 5.9%. Wagner concluded that listening played an important part in teaching competence and TOEFL iBT listening scores should be used for ITA screening purposes.

### Summing Up

As is shown above, 87 studies in our dataset were conducted to explore L2 listening assessment from a wide range of perspectives, tapping into 13 research themes in relation to the six components of the socio-cognitive framework. The vast majority of the studies explored test-taker characteristics, cognitive validity, context validity, and scoring validity, accounting for 94.25%. As important variables influencing listening test scores, a variety of test-taker characteristics were investigated. Research on cognitive validity examined items targeting different listening subskills and levels of listening process. Various research methods were used to uncover the complex cognitive processes, with innovative technology used to investigate test-takers’ eye movement and brain activation patterns. In terms of context validity, task setting, linguistic demands (task input and output), and speakers have received considerable attention. Three parameters (i.e., item bias, internal consistency, and grading and awarding) influencing the scoring validity of L2 listening assessment were explored. In comparison, there is a small number of studies focusing on consequential validity and criterion-related validity, with only one study addressing the issue of test washback, and three studies exploring criterion-related validity. While helping to deepen our understanding of listening assessment from different perspectives, this review also brings to light many questions that need to be answered and a large amount of work that needs to be done.

## Discussion and Recommendations For Future Research

Findings of the present study suggest that more research efforts are needed in the field of L2 listening assessment. Recommendations for future research are discussed below from two perspectives, one on the four components which have been extensively investigated and the other on the two components which did not receive much attention (i.e., consequential validity and criterion-related validity).

Although research on physical/physiological characteristics underscores the importance of understanding test-takers’ special needs, it is challenging to accommodate test takers with special needs, since it is not clear how test fairness and validity are affected by providing special arrangements for a particular group of test takers. In relation to experiential characteristics, the effect of test preparation was explored, indicating that test-takers’ familiarity with the test format and preparation for listening tests are important variables influencing test performance. Future research should consider the role of test-takers’ listening proficiency in test preparation. Moreover, with young learners constituting a large proportion of language learners, more studies are needed to explore the physical/physiological, psychological, and experiential characteristics of young test takers.

Studies on cognitive validity revealed that L2 listening is a complicated and dynamic cognitive operation. Moreover, research on L2 listening subskills and levels of comprehension indicates that it is challenging for test developers to operationalize the construct of L2 listening systematically. Recent years have witnessed an increasing use of advanced technology, such as eye-tracking technology and neuroimaging, which has brought about important development in the field. For instance, the notion of cognitive validity has been expanded, as researchers probed into the neurocognitive mechanism of test takers (Aryadoust et al., 2020). However, it is still difficult to understand test-takers’ cognitive processes due to the highly overlapping and synergistic nature of comprehension (Alderson, 1990). For instance, test takers may simultaneously use higher-level and lower-level processing to comprehend the input (Brindley, 1998), and it is challenging to distinguish different levels of processing. Therefore, the authors think that research on cognitive processes is an important area where new perspectives are still unfolding and more research is needed to elucidate the relationship between cognitive processes and listening performance.

It is not surprising that a high proportion of studies investigated context validity since test developers should design tasks and adjust task characteristics that can retain key features of language use contexts and the way test tasks are designed and controlled has a direct effect on test authenticity (Bachman, 1990). Despite the abundance of research on context validity, the authors think that more efforts should be made to increase task authenticity and to avoid construct under-representation and construct irrelevance. As discussed previously, the use of visuals in listening assessment improves task authenticity as real-life listening usually involves visual input, but it may introduce construct-irrelevant variance if the test is designed to assess test-taker’ mastery of the language itself. Similarly, whether to incorporate varieties of accents remains open for discussion. The use of diverse accents in L2 listening tests resembles the real-life context which requires multidialectal listening ability, but certain test takers may be advantaged due to the shared-L1 effect, which raises concerns about test fairness. Therefore, more research is needed to elucidate the shared-L1 effect and justify the use of non-standard accents in listening assessment.

In relation to scoring validity, the theme of grading and awarding warrants more research endeavors. With descriptive and interpretable score reporting required for improving instructional designs and guiding students’ learning (Alderson, 2005; Jang, 2008), more meaningful descriptors should be attached to listening scores. Future studies can consider providing richer and more detailed feedback of listening assessment for test users and convert test scores to plausible statements about test-takers’ listening ability (Taylor and Geranpayeh, 2013). Also, more research is needed to explore the utility of feedback for L2 listening test users, including learners, teachers, and institutions.

The following are some recommendations for future research on the two components which did not receive much attention in our dataset, i.e., consequential validity and criterion-related validity. Consequential validity is one of the key areas for future research, and themes of test use, consequences, test fairness, and ethics warrant more research efforts, given that test washback and impact have become major areas of study in the field of language testing (Alderson, 2004). As Shohamy (2007, p. 117) pointed out, “the quality of tests is not judged merely by their psychometric traits but rather in relation to their impact, ethicality, fairness, values, and consequences.”

There is a scarcity of research on the washback and impact of listening tests in our dataset, probably due to the complex mechanism of washback and impact in different social and educational contexts (Alderson and Wall, 1993; Hawkey, 2013). Washback and impact are affected by simply changing test methods and educational contexts (Cheng, 1997; Alderson, 2004) and may be independent of the original intentions of the test developers (Cheng et al., 2004). Therefore, the investigation of test washback and impact is time-consuming and complicated by a wide range of variables influencing learning and teaching, which requires a long-term and relatively complicated research program (Alderson and Wall, 1993; Nguyen and Gu, 2020). Furthermore, the study of washback and impact in the field of L2 listening assessment is more challenging due to the complexity of listening construct (Hawkey, 2013).

More research efforts are needed to explain the mechanism of washback and impact of L2 listening tests with education innovation and change in various contexts. The study of test washback and impact should be situated within the micro contexts (e.g., the school setting) and macro contexts (e.g., the sociocultural environment where the test is used; Cheng L., 2004). Considering the rapid change in educational policy and the needs of stakeholders, a better understanding of how the washback and impact of L2 listening tests occur is needed. In addition, with the increasingly widespread use of high-stakes tests that have important consequences for individuals and institutions (Bailey, 1999; Alderson, 2004; Green, 2013), future research should investigate the washback and impact of high-stakes listening tests.

In addition to consequential validity, criterion-related validity is also important with the development of language proficiency scales, such as the Common European Framework of Reference for languages: Learning, Teaching, Assessment (CEFR) and the recently released China’s Standards of English Language Ability (CSE). One of the aims of these proficiency scales is to promote communication between researchers and practitioners in the fields of language learning, teaching, and assessment (Council of Europe, 2001; National Education Examinations Authority, 2018). Although aligning tests to proficiency scales is conducive to bridging the gap between learning and assessment, the procedure of alignment is complex (Harsch and Rupp, 2011). Thus, future research is needed to provide evidence for the validity of using these proficiency guidelines for listening assessment.

## Conclusion

In the present study, a review of research on L2 listening assessment was conducted using Weir’s (2005) socio-cognitive framework. With a total of 87 studies collected, 13 research themes were identified in relation to the six components of the framework and analyzed. Recommendations for future research in the field were discussed from the perspectives of the four components that were extensively investigated and the other two components which did not receive much attention in our dataset, that is, consequential validity and criterion-related validity. While trying to give a comprehensive review of relevant research, the authors are fully aware of the limitations of the present study. For one thing, only studies from 14 peer-reviewed journals and two research report series were reviewed, and research on L2 listening assessment published in other journals, research report series, conference proceedings, or book series were not included due to time and space limit. For another, studies written in languages other than English were not included as a result of resource and space constraints. Despite the limitations mentioned above, this study provides valuable insights into various factors that can influence test-takers’ performance in L2 listening assessment and sheds light on the state-of-the-art research in L2 listening assessment.

## Author Contributions

LH designed the study, coded the data, and drafted the manuscript. ZJ collected the data, coded the data, and drafted the manuscript together with LH. All authors contributed to the article and approved the submitted version.

### Conflict of Interest

The authors declare that the research was conducted in the absence of any commercial or financial relationships that could be construed as a potential conflict of interest.
